# Role of Probiotics in the Treatment of Nonalcoholic Fatty Liver Disease: A Meta-analysis

**DOI:** 10.5005/jp-journals-10018-1233

**Published:** 2017-09-29

**Authors:** Anurag S Lavekar, Dhananjay V Raje, Tanuja Manohar, Amarja A Lavekar

**Affiliations:** 1 Department of Gastroenterology and Hepatology, JSS Hospital, Mysuru, Karnataka, India; 2 Department of Data Analysis Group, MDS Bio-Analytics Private Limited, Nagpur, Maharashtra, India; 3 Department of Medicine, NKP Salve Institute of Medical Sciences & Research Center, Nagpur, Maharashtra, India; 4 Department of Radiology, Triveni Hospital, Nanded, Maharashtra, India

**Keywords:** Heterogeneity, Meta-analysis, Probiotics, Ultrasonographic grade.

## Abstract

**Aim:**

Despite extensive ongoing research, there is scarcity of widely accepted therapeutic options for the treatment of nonalcoholic fatty liver disease (NAFLD). Probiotics are a promising treatment option for treating NAFLD; however, their effectiveness needs to be established. Since any single randomized controlled trial (RCT) cannot establish the role of probiotics in the treatment of NAFLD, this study aims at meta-analysis of different RCTs.

**Materials and methods:**

Extensive search was done by two independent observers for RCTs studying the role of probiotics in the treatment of NAFLD. The parameters under consideration were body mass index (BMI), aspartate aminotransferase (AST), alanine aminotransferase (ALT), homeostatic model assessment of insulin resistance (HOMA-IR), serum triglycerides (TGs), and ultrasonographic grades of fatty liver. Jadad scale was used to select the articles for meta-analysis. Heterogeneity in the results was evaluated using chi-square test and *I*^2^. Significant heterogeneity in the results was decided based on p-value < 0.05 and the corresponding *I*^2^ close to 0%.

**Results:**

Seven studies qualified for meta-analysis. Use of probiotics significantly caused reduction in BMI (p < 0.0001), ALT (p < 0.0001), AST (< 0.0001), HOMA-IR (p = 0.006), and ultrasonographic grade of fatty liver (p = 0.0051). Heterogeneity in other parameters was contributed mainly by couple of previous studies.

**Conclusion:**

Meta-analysis shows that variety of parameters has significant improvement after probiotic treatment in different RCTs. However, the magnitude of improvement is not uniform across studies due to varying strains, dose patterns, and treatment duration. In future, probiotics remain a promising option for treating NAFLD.

**How to cite this article:** Lavekar AS, Raje DV, Manohar T, Lavekar AA. Role of Probiotics in the Treatment of Nonalcoholic Fatty Liver Disease: A Meta-analysis. Euroasian J Hepato-Gastroenterol 2017;7(2):130-137.

## INTRODUCTION

There is a consensus regarding increasing worldwide prevalence of obesity and hence, NAFLD and its impact on health, especially the progression to cirrhosis of liver and hepatocellular carcinoma. The prevalence of NAFLD ranges from 9 to 40% and varies across different regions.^1-4^ Since it is an obesity-related disorder, the main emphasis of treatment for NAFLD has been on exercise and weight reduction so far. Musso et al^5^ demonstrated improvement in liver histology with reduction in weight. However, quite often, it is difficult to practice it in real life, and thus there is always scope for exploring newer therapeutic strategies. Metformin, vitamin E, statins, pioglitazone, ursodeoxycholic acid, probucol, N-acetyl cysteine, low-dose carnitine, and pentoxifylline are some of the studied therapeutic options available. But each has its own limitations to be used on a wide scale and nothing can be said conclusively about their efficacy.^6,7^

Probiotics for the treatment of NAFLD are seemingly a promising treatment option. Relatively easy availability, low cost, and absence of serious side effects make probiotics a lucrative choice.

In animal studies, probiotics have profound role in improvement of nonalcoholic steatohepatitis (NASH).^8-10^ Probiotics are defined as live microorganisms, which when consumed in adequate amounts, confer health effects on the host.^11^ Probiotics supposedly delay disease progression and prevent complications by modulating intestinal flora, intestinal permeability, and inflammatory response.^12^

Since a single randomized clinical trial cannot establish or downplay the efficacy of probiotics for treating NAFLD, this study aims at systematic reviewing of the multiple RCTs involving use of probiotics for the treatment of NAFLD.

## MATERIALS AND METHODS

### Data Collection

Two independent observers searched PubMed, Cochrane, Embase full text data base with NAFLD, NASH, probiotics, symbiotic as keywords during January to February 2016. Extensive search was conducted, which included studies like randomized clinical trials, comparative studies, etc. The parameters of interest were BMI, AST, ALT, HOMA-IR, TGs, and ultrasonographic grade of fatty liver. Jadad scale was used to select articles for meta-analysis. Each article was assessed on criteria like randomization methods used, blinding, and follow-up, as suggested by Jadad. All the selected articles had score >3 and accordingly were retained for downstream analysis.

### Statistical Analysis

The key biochemical parameters of NAFLD measured on real scale and expressed in terms of mean and standard deviation in different studies were considered. Heterogeneity in the results was evaluated using Chi-square test and *I*^2^. Statistically significant heterogeneity in the results was decided based on p-value <0.05 and corresponding *I*^2^ close to 0%. The radiological parameter, i.e., grade of NAFLD, expressed in terms of frequencies by different researchers was considered for evaluation. Odds ratios were obtained for different parameters and accordingly the corresponding heterogeneity was referred to decide upon the heterogeneity of outcomes across studies. Fixed effects model was referred to arrive at the significance of overall effect. All the analyses were performed using NCSS 2007 software.

**Table 1:** **Table 1:** Studies included in meta-analysis*

*Reference*		*Sample size*		*Diagnostic method*		*Intervention*		*Duration (months)*	
Vajro et al^13^		20		Histological		*Lactobacillus rhamnosus* strain GG in pediatric obesity-related liver disease		2	
Aller et al^14^		30		Histological		*Lactobacillus bulgaricus* and *Streptococcus thermophilus vs* placebo		3	
Malaguarnera et al^15^		66		Histological		*Bifidobacterium longum* + Fos *vs* placebo		6	
Wong et al^16^		20		Histological		Lepicol probiotic and prebiotic formula *vs* nothing		6	
Shavakhi et al^17^		64		Histological/radiological		Probiotic and metformin on liver aminotransferases in NASH		6	
Alisi et al^18^		44		Histological		The beneficial effects of VSL#3 in obese children with NASH		4	
Eslamparast et al^19^		52		Histological		Symbiotic supplementation in NAFLD		7	

*All studies were double-blind and had follow-ups

## RESULTS

The flow for selection of studies is depicted in Flow Chart 1. Out of 19 relevant studies published during the period 2005 to 2015, only 7 studies qualified for meta-analysis considering the study parameters, statistical outcome measures, and the probiotic interventions. Remaining 12 studies were ignored from the analysis. Table 1 provides the brief description of selected studies.^13-19^ The selected studies ranged between 2011 and 2015 and mostly target biochemical and radiological parameters with probiotic intervention. The duration of studies ranged between 2 and 7 months. All studies were double-blinded with follow-ups and had proper matching of baseline characteristics between treatment groups. The analysis of different biochemical parameters was performed across studies following the approach described in methods and the results are shown in Table 2.

**Fig. 1:**
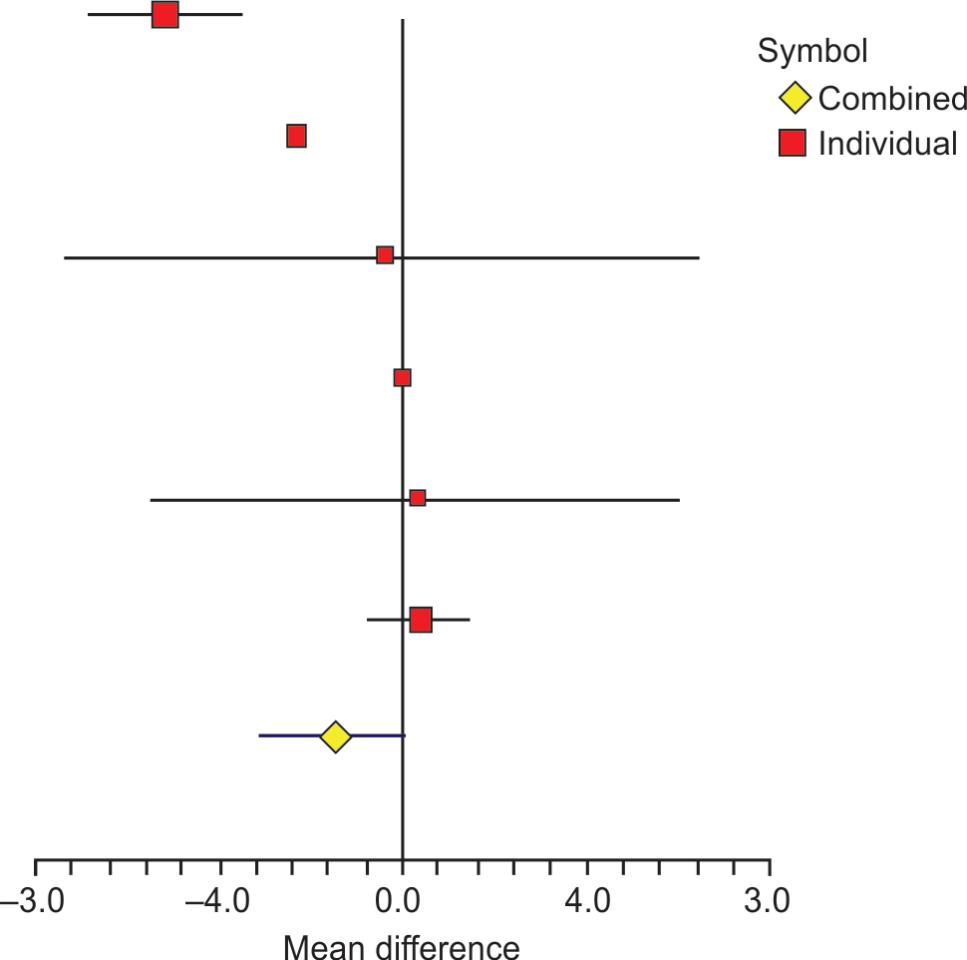
Forest plot showing the effect of probiotics on BMI in different studies

There were six studies that reported BMI of samples for experimental and control groups in terms of mean and standard deviation (Table 2). All the studies mentioned about the change in the BMI from baseline. The overall difference in the experimental and control groups of mean change was significant with a weighted mean difference of -1.45 (95% confidence interval (CI): -3.06, 0.16) and associated p-value <0.0001. The heterogeneity across studies was significant as revealed by *I*^2^ of 97.48% with p-value <0.0001. A forest plot of mean difference of BMI is shown in Figure 1. The heterogeneity was mainly contributed by the studies of Shavakhi et al^17^ and Alisi et al,^18^ which showed a large change in the BMI, i.e., -2.2 and -5.2 kg/m^2^ respectively, in the experimental group compared with other studies. If these two studies are excluded, remaining four studies showed significant homogeneity, as also reported by Ma et al.^20^ The resulting *I*^2^ value was 0% with corresponding p-value of 0.9407.

**Table 2:** **Table 2:** Comparison of BMI across different studies

		*Experiment*						*Control*						*Difference*							
*Body mass index*		*Before*		*After*				*Before*		*After*				*Experiment*		*Control*				*Mean difference*	
		*Mean (SD)*		*Mean (SD)*		*Total*		*Mean (SD)*		*Mean (SD)*		*Total*		*Mean (SD)*		*Mean (SD)*		*Weight*		*Random, 95% CI*	
Vajro et al^13^		2.29 (0.3)		2.21 (0.31)		10		2.12 (0.24)		2 (0.26)		10		–0.08 (0.42)		–0.12 (0.33)		23.83		0.04 (-0.31, 0.39)	
Aller et al^14^		30.2 (4.5)		31.1 (4.8)		14		29.5 (5.5)		30.1 (6.1)		14		0.9 (6.51)		0.6 (8.25)		6.33		0.30 (-5.47, 6.07)	
Malaguarnera et al^15^		27.3 (1.4)		26.4 (1.8)		34		27.2 (1.3)		25.9 (1.9)		32		–0.9 (2.31)		–1.3 (2.29)		21.61		0.40 (-0.73, 1.53)	
Wong et al^16^		30.2 (5)		29.3 (4.3)		10		28.7 (5.7)		28.2 (5.6)		10		–0.9 (6.61)		–0.5 (8.05)		4.96		–0.40 (-7.32, 6.52)	
Alisi et al^18^		27.1 (0.01)		24.9 (0.2)		22		25.6 (0.01)		25.7 (0.24)		22		–2.2 (0.20)		0.1 (0.24)		24.03		–2.30 (-2.43, -2.17)	
Shavakhi et al^17^		28.6 (2)		23.4 (2.3)		31		28.2 (2.5)		28.16 (2.6)		32		–5.2 (3.05)		–0.04 (3.58)		19.25		–5.16 (-6.84, -3.48)	
Total						121						120						100		–1.45 (-3.06, 0.16)	

Heterogeneity: χ^2^ = 198.98, DF = 5 (p = 0.00001), *I*^2^ = 97.48%; overall effect (p < 0.0001); SD: Standard deviation

**Flow Chart 1:**
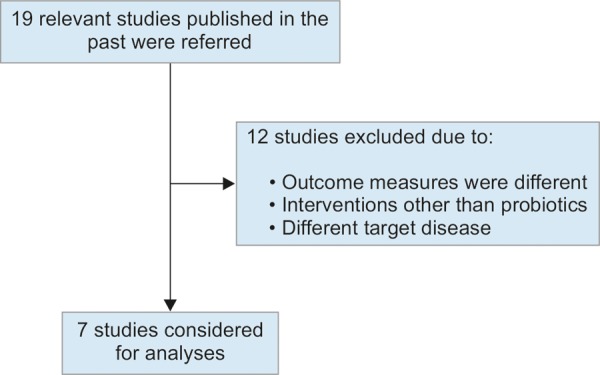
Selection of studies

**Fig. 2:**
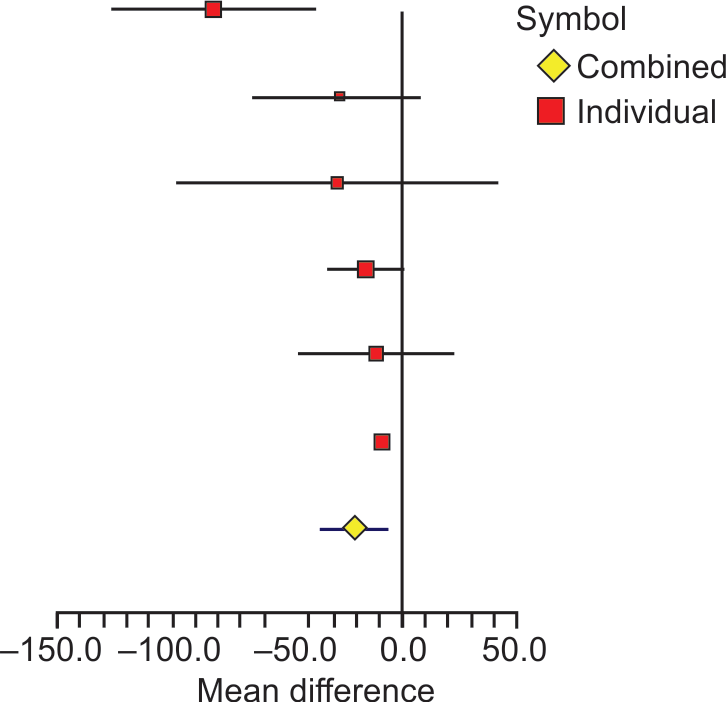
Forest plot showing the effect of probiotics on ALT in different studies

The ALT was reported by same six studies as shown in Table 3. The overall difference of mean change in the experimental and control groups was significant with weighted mean difference of -20.97 (95% CI: -36.14, -5.81) and with p-value < 0.0001. The heterogeneity across studies was significant as observed through *I*^2^ of 61.47% with p-value of 0.0236. A forest plot of mean difference of ALT in different studies is shown in Figure 2. The heterogeneity was due to study by Shavakhi et al,^17^ which showed the largest mean difference of -82.6 (95% CI: -127.22, -37.98). If the study is excluded, the resulting *I*^2^ becomes 0% with p-value of 0.9407, indicating absence of heterogeneity or strong homogeneity.

**Fig. 3:**
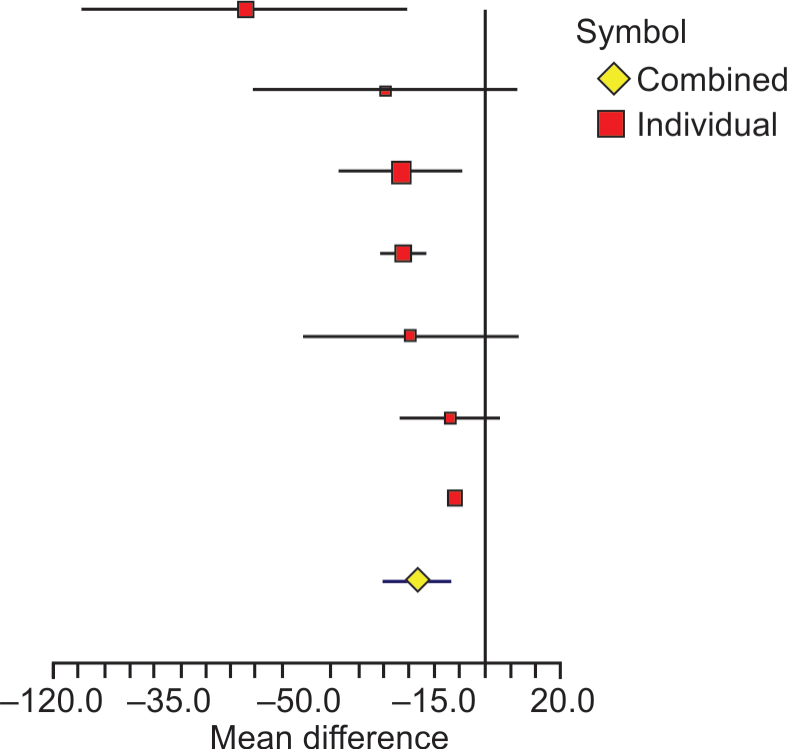
Forest plot showing the effect of probiotics on AST in different studies

Another key biochemical parameter AST was studied by seven researchers in last 5 years (Table 4). The overall difference of mean change in experimental and control groups was significant, with weighted mean difference of -19.24 (95% CI: -28.75, -9.74) and with p-value < 0.0001. The heterogeneity in the studies was highly significant with *I*^2^ value of 78.36% and the corresponding p-value < 0.0001. The heterogeneity of results was mainly due to studies by Shavakhi et al^17^ and Alisi et al.^18^ Excluding these, remaining five studies showed strong homogeneity with *I*^2^ value of 0% and the corresponding p-value of 0.5468. A graphical representation of differences in studies has been shown through forest plot in Figure 3.

**Fig. 4:**
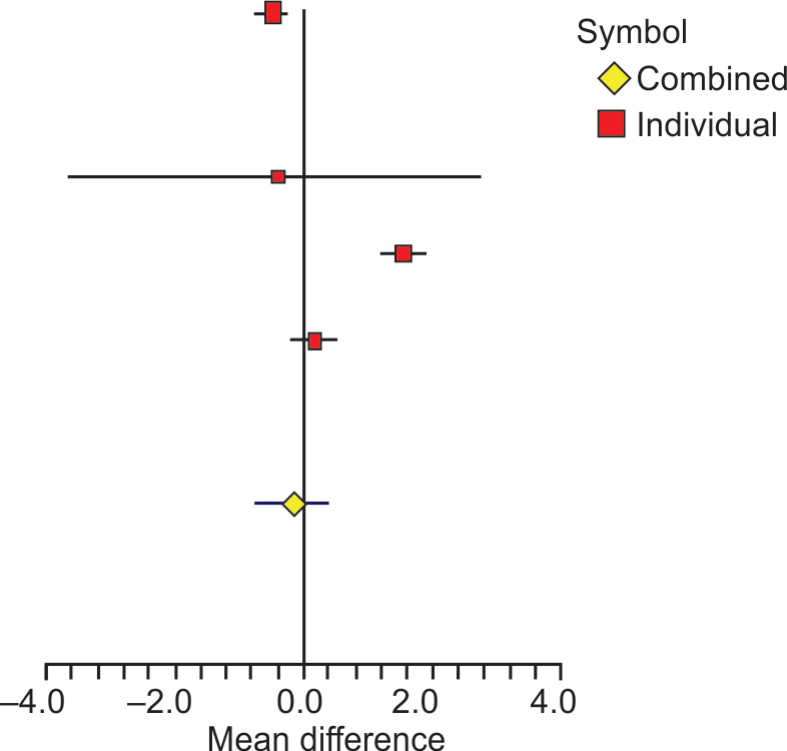
Forest plot showing the effect of probiotics on HOMA-IR in different studies

The HOMA-IR was also compared across different studies (Table 5). The overall difference of mean change in two groups was significant with weighted mean difference of -0.15 (95% CI: -0.74, 0.44) and the corresponding p-value of 0.006. The parameter showed significant heterogeneity across studies with *I*^2^ of 76.79% and p-value of 0.0134. A forest plot representation showing mean difference of parameter across studies is given in Figure 4. Study by Alisi et al^18^ contributed heterogeneity to the parameter, while the other two studies showed strong homogeneity as also mentioned by Ma et al.^20^

**Table 3:** **Table 3:** Comparison of ALT across different studies

		*Experiment*						*Control*						*Difference*							
		*Before*		*After*				*Before*		*After*				*Experiment*		*Control*				*Mean difference*	
*ALT*		*Mean (SD)*		*Mean (SD)*		*Total*		*Mean (SD)*		*Mean (SD)*		*Total*		*Mean (SD)*		*Mean (SD)*		*Weight*		*Random, 95% CI*	
Vajro et al^13^		70.3 (34.76)		40.1 (22.37)		10		63.6 (18.47)		61.6 (31.8)		10		–30.2 (41.31)		–2 (36.44)		12.67		–28.20 (-64.78, 8.40)	
Aller et al^14^		67.7 (25.1)		60.4 (30.4)		14		60.7 (32.1)		64.8 (35.5)		14		–7.3 (39.20)		4.1 (47.69)		13.57		–11.40 (-45.31, 22.51)	
Malaguarnera et al^15^		101 (24.7)		47.1 (19.8)		34		96.1 (24.2)		58.1 (27.2)		32		–53.9 (31.18)		–38 (36.88)		25		–15.90 (-32.66, 0.86)	
Wong et al^16^		96 (75)		71 (52)		10		72 (30)		75 (44)		10		–25 (91.07)		3 (53.40)		4.664		–28.00 (-98.14, 42.14)	
Alisi et al^18^		34 (1)		33 (1)		22		42 (1)		50 (5)		22		–1 (1.41)		8 (5.12)		35.13		–9.00 (-11.28, -6.72)	
Shavakhi et al^17^		133.7 (70)		45.2 (32.5)		31		118.4 (67.9)		112.5 (68.7)		32		–88.5 (77.87)		–5.9 (97.77)		8.975		–82.60 (-127.22, -37.98)	
Total						121						120						100		–20.97 (-36.14, -5.81)	

Heterogeneity: χ^2^ =12.98; DF = 5 (p = 0.0236), *I*^2^ = 61.47%; overall effect (p < 0.0001); SD: Standard deviation

**Table 4:** **Table 4:** Comparison of AST across different studies

		*Experiment*						*Control*						*Difference*							
		*Before*		*After*				*Before*		*After*				*Experiment*		*Control*				*Mean difference*	
*AST*		*Mean (SD)*		*Mean (SD)*		*Total*		*Mean (SD)*		*Mean (SD)*		*Total*		*Mean (SD)*		*Mean (SD)*		*Weight*		*95% CI*	
Aller et al^14^		41.3 (15.5)		35.6 (10.4)		14		31.7 (13.1)		36.4 (13.8)		14		–5.7 (18.70)		4.7 (19.13)		17.02		–10.40 (-25.02, 4.23)	
Vajro et al^13^		70.3 (34.76)		40.1 (22.37)		10		63.6 (18.47)		61.6 (31.8)		10		–30.2 (41.40)		–2 (36.40)		6.08		–28.20 (-64.82, 8.42)	
Malaguarnera et al^15^		109 (23.2)		39.4 (28.2)		34		107.1 (21.4)		61.2 (25.4)		32		–69.6 (36.89)		–45.9 (33.29)		14.45		–24.00 (-41.32, -6.68)	
Wong et al^16^		50 (25)		37 (20)		10		38 (15)		46 (27)		10		–13 (31.93)		8 (31.36)		8.17		–21.00 (-50.73, 8.73)	
Alisi et al^18^		34 (1)		33 (1)		22		42 (1)		50 (5)		22		–1 (1.40)		8 (5.11)		26.47		–9.00 (-11.28, -6.72)	
Eslamparast et al^19^		66.38 (2.6)		35.05 (2.7)		26		68.29 (9.41)		60.34 (13.1)		26		–31.33 (3.73)		–7.95 (16.02)		24.00		–23.38 (-29.86, -16.90)	
Shavakhi et al^17^		123.1 (72)		44.2 (33.9)		31		125.3 (71)		113.4 (71)		32		–78.9 (79.91)		–11.9 (99.44)		3.88		–67.00 (-112.54, -21.46)	
Total						147						146						100		–19.24 (-28.75, -9.74)	

Heterogeneity: χ^2^ = 27.64; DF = 6 (p = 0.0001), *I*^2^ = 78.36%; overall effect (p < 0.0001); SD: Standard deviation

**Table 5:** **Table 5:** Comparison of HOMA-IR across different studies

		*Experiment*						*Control*						*Difference*							
		*Before*		*After*				*Before*		*After*				*Experiment*		*Control*				*Mean difference*	
*HOMA-IR*		*Mean (SD)*		*Mean (SD)*		*Total*		*Before mean (SD)*		*After mean (SD)*		*Total*		*Mean (SD)*		*Mean (SD)*		*Weight*		*Random, 95% CI*	
Aller et al^14^		4.5 (2.6)		4.2 (2.4)		14		4.2 (3.2)		4.3 (3.4)		14		–0.3 (3.55)		0.1 (4.66)		3.51		–0.40 (-3.62, 2.82)	
Malaguarnera et al^15^						34						32		–1.1 (0.52)*		–0.64 (0.6)*		49.84		–0.46 (-0.74, -0.18)	
Alisi et al^18^		4.3 (0.3)		3.3 (0.3)		22		4.7 (0.4)		3.5 (0.6)		22		–1 (0.42)		–1.2 (0.72)		46.66		0.20 (-0.16, 0.56)	
Total						70						68						100		–0.15 (-0.74, 0.44)	

*Ma et al^20^; heterogeneity: χ^2^ = 8.62; DF = 2 (p = 0.0134), *I*^2^ = 76.79%; overall effect (p = 0.006); SD: Standard deviation

**Table 6:** **Table 6:** Comparison of TG across different studies

		*Experiment*						*Control*						*Difference*						*Mean difference*	
		*Before*		*After*				*Before*		*After*				*Experiment*		*Control*				*Random 95% CI*	
*TG*		*Mean (SD)*		*Mean (SD)*		*Total*		*Mean (SD)*		*Mean (SD)*		*Total*		*Mean (SD)*		*Mean (SD)*		*Weight*			
Aller et al^14^		171.1 (95.4)		150.9 (61.1)		14		134.8 (51.8)		147.2 (48.6)		14		–20.2 (113.24)		12.4 (71.14)		31.33		–32.60 (-106.06, 40.86)	
Alisi et al^18^		99 (4)		110 (9)		22		98 (3)		102 (10)		22		11 (9.90)		4 (10.46)		35.84		7.00 (0.80, 13.17)	
Shavakhi et al^17^		260.5 (100)		149.7 (57)		31		242.5 (87)		188.7 (68.9)		31		–110.8 (114.29)		–53.8 ( 110.26)		32.84		–164.60 (-221.65, -107.55)	
Total						67						67						100		–61.75 (-171.84, 48.33)	

Heterogeneity: χ^2^ = 36.88; DF = 2 (p = 0.00001), *I*^2^ =94.57%; overall effect (p = 0.1175); SD: Standard deviation

**Fig. 5:**
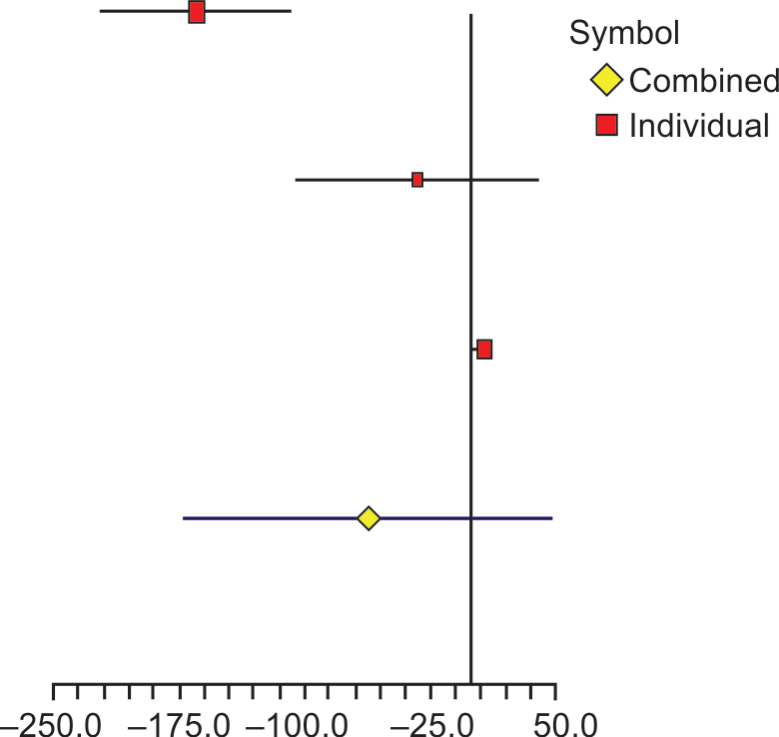
Forest plot showing the effect of probiotics on TG in different studies

Similarly, TG was also analyzed in three studies as shown in Table 6. The overall difference of mean change between experimental and control groups was insignificant with weighted mean difference of -61.75 (95% CI: -171.84, 48.33) with associated p-value of 0.1175. The *I^2^* value obtained was 94.57%, indicating significant heterogeneity across studies with a p-value < 0.0001. A graphical representation of heterogeneity has been shown through mean differences of TG in different studies (Fig. 5). It is quite evident that Shavakhi et al^17^ study contributed most to the heterogeneity of the parameter. Without this study, remaining two showed lower heterogeneity with *I^2^* value of 17.93% and with p-value of 0.2696.

After 2013, there are no studies reporting about few more important indicators of NAFLD like tumor necrosis factor-a, total cholesterol, low-density lipoprotein, high-density lipoprotein, and glucose. Ma et al^20^ had already discussed about these parameters with previous studies.

**Table 7:** **Table 7:** Comparison of normal grade of NAFLD in two studies

*Normal grade*		*Experimental*		*Control*		*Odds ratio (OR)*		*OR: 95% CI*		*Weight*	
Shavakhi et al^17^		12/31		2/32		7.8205		(1.7945, 34.0821)		80.1906	
Alisi et al^18^		5/22		0/22		14.1429		(0.7316, 273.3901)		19.8094	
Total						8.7944		(2.3536, 32.8613)		100	

Heterogeneity: χ^2^ = 0.1233; DF = 1 (p = 0.7255); *I*^2^ = 0%; SD: Standard deviation

**Fig. 6:**
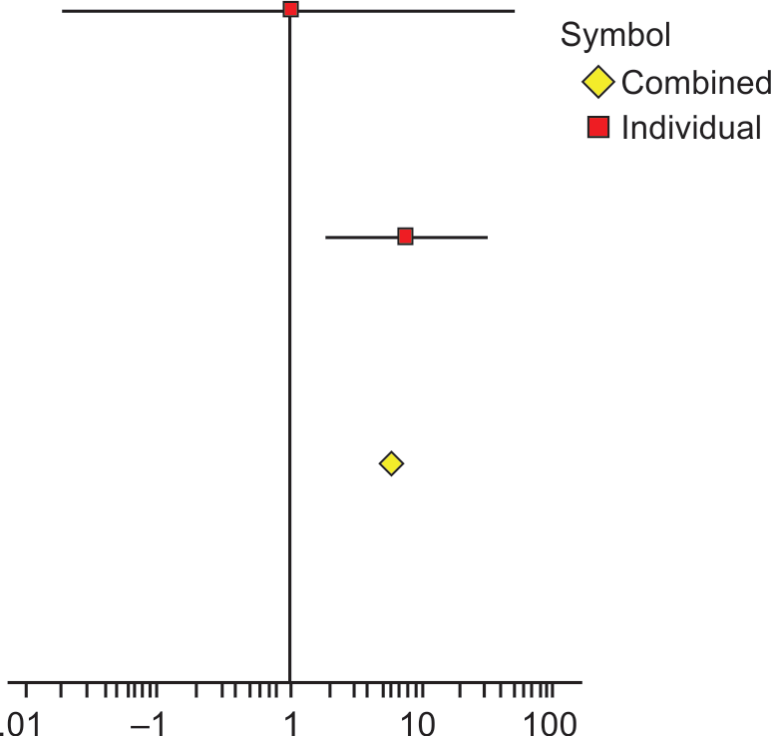
Forest plot showing the effect of probiotics on NAFLD grade observed through ultrasound

### Radiological Parameters

The grade of NAFLD obtained through ultrasound was also studied through meta-analysis. Two studies (Table 7) reported ultrasound appearances and classified patients at baseline into normal, low, moderate, and severe categories. Posttreatment, the same set of patients were reclassified to assess the effect of treatment modalities. The event of interest was "how many patients attained *normal* grade, posttreatment"; accordingly, a 2-by-2 contingency matrix was obtained for each study to arrive at the odds ratio, as a measure of treatment effect. Table 7 shows the odds ratio for the two studies and corresponding 95% CI. The combined odds ratio obtained was 8.7944 (95% CI: 2.3536, 32.8613) with p-value of 0.0051, implying at least one study with nonzero treatment effect. The heterogeneity test resulted into *I^2^* value of 0% with corresponding p-value of 0.7255, indicating homogeneity of effect between two studies. A forest plot showing the effect of probiotics on grade of NAFLD using ultrasound is shown in Figure 6.

## DISCUSSION

As liver receives its majority blood supply through portal vein, there is a constant anatomical and functional relationship between gut and liver and this is based on the gut-liver axis.^21^ It is logical that any alterations in the gut homeostasis affect the liver as well. It is increasingly evident that malfunctioning of this gut-liver axis, i.e., intestinal dysbiosis, small intestinal bacterial overgrowth, and increased intestinal permeability or leaky gut have a role in development and progression of NAFLD.^22-25^ There is a definite role of normal gut microbiota in development of intestinal immunity.^21^ This normal gut microbiota is altered in obese and NAFLD patients. Composition of intestinal microbiota in normal and in obese and NAFLD individuals and its detailed discussion is beyond the scope of this work. Changes in the normal gut microbiota bring about liver inflammation.^21^ Supplementing a patient of NAFLD with probiotics aims at restoration of normal gut flora and thereby reducing liver inflammation. This forms the rationale for treating NAFLD patients with probiotics.

Majority of the studies published in the past showed that parameters like BMI, ALT, AST, HOMA-IR, TGs, and liver radiology differed significantly between experimental (those who received probiotics) and control group. Meta-analysis of these parameters across studies also revealed significant variability (heterogeneity) as indicated by the corresponding *I^2^* values. In particular, studies by Shavakhi et al^17^ and Alisi et al^18^ significantly influenced the heterogeneity of parameters as evident from Forest plots. Alisi et al^18^ have used eight different strains of bacteria in children. Shavakhi et al^17^ have compared use of metformin in combination with probiotics *vs* metformin alone and its impact on liver inflammation. They found that metformin when used in combination with probiotics was significantly better than metformin alone in reducing liver inflammation. Metformin itself is one of the therapeutic tools for treating NAFLD. It is difficult to comment on the precise cause of heterogeneity due to these two studies.

Eliminating these two data sets from meta-analysis resulted in improved homogeneity of all parameters, although limiting the number of case studies in the analysis. In other words, the mean difference of parameters between experimental and control groups was statistically similar across studies, except the above two studies. However, these were the only two studies reporting on sonographic grades of NAFLD in patients. There was significant homogeneity in the outcomes as regards the normal grade cases. The radiological evaluations showed consistency in these two studies; however, the number of studies till date is very scarce to strongly comment on the homogeneity.

This meta-analysis has certain limitations. Despite increase in the number of RCTs determining efficacy of probiotics for treating NAFLD, there is no uniformity in the parameters studied with a few exceptions like BMI and ALT. There is hardly any study describing the effect of probiotics on waist circumference and waist-hip ratio, which are closely related to NAFLD. There was only one study reporting biopsy-proven efficacy of probiotics in the treatment of NAFLD, which is the gold standard for diagnosis of NAFLD even today.^20^ The sensitivity of ultrasonography in detecting fatty liver ranges from 60 to 94% and also depends on the degree of steatosis, i.e., cannot identify the fatty infiltration of the liver below 30%.^4,20,26^ By definition, fatty liver has more than 5% hepatocytes containing fat or more than 5% of liver weight comprising of fat.^27^ Magnetic resonance imaging is by far superior to ultrasound examination in this regard, which can diagnose hepatic steatosis with a lower limit of 3%.^28^ Only Wong et al^16^ have attempted measurement of intrahepatic triglyceride content.

The issue of interobserver variability when ultrasound examination is used for diagnosing NAFLD and determining the efficacy of probiotics for treating NAFLD is not overcome yet.^29,30^ To add to this limitation, we could retrieve only two studies using USG for the purpose.^17,18^

There is a serious need for consensus regarding type, dose, and duration of probiotics. All these limitations point toward the need of more and more RCTs. It is mandatory to address the above-mentioned limitations as these need to be overcome in upcoming trials. There are ample animal studies regarding usefulness of probiotics in reducing liver inflammation through diverse mechanisms.^8-10,31-35^ A study done on animal model by Yalçin et al^36^ reported improvements in histological grades, steatosis, and ballooning scores but worsened triglyceridemia. So far, each individual human study reported in favor of use of probiotics in treating NAFLD. Ma et al^20^ in their meta-analysis also encouraged the use of probiotics for treating NAFLD, but only based on four studies. To the best of our knowledge, there is not a single study from India where lactobacilli (probiotic bacteria)-containing food products, such as curd, buttermilk, etc., that are contents of a regular meal mentioning about the role of probiotics in treatment of NAFLD. Probiotics remain a promising treatment option for NAFLD. Even though there is increasing amount of evidence for a role of probiotics in treating NAFLD, more work in this direction is needed to establish their role. Given the easy availability, low cost of therapy, relative paucity of major adverse effects, and the limitations not that difficult to overcome, we do not see any hassles for more number of RCTs across the world.
